# TGF-β1 Signaling Impairs Metformin Action on Glycemic Control

**DOI:** 10.3390/ijms25042424

**Published:** 2024-02-19

**Authors:** Quan Pan, Weiqi Ai, Shaodong Guo

**Affiliations:** Department of Nutrition, College of Agriculture and Life Sciences, Texas A&M University, College Station, TX 77843, USA; quan.pan@ag.tamu.edu (Q.P.); awq123007@tamu.edu (W.A.)

**Keywords:** TGF-β1, metformin, AMPK, Foxo1, LY2157299, hepatic glucose production

## Abstract

Hyperglycemia is a hallmark of type 2 diabetes (T2D). Metformin, the first-line drug used to treat T2D, maintains blood glucose within a normal range by suppressing hepatic glucose production (HGP). However, resistance to metformin treatment is developed in most T2D patients over time. Transforming growth factor beta 1 (TGF-β1) levels are elevated both in the liver and serum of T2D humans and mice. Here, we found that TGF-β1 treatment impairs metformin action on suppressing HGP via inhibiting AMPK phosphorylation at Threonine 172 (T172). Hepatic TGF-β1 deficiency improves metformin action on glycemic control in high fat diet (HFD)-induced obese mice. In our hepatic insulin resistant mouse model (hepatic insulin receptor substrate 1 (IRS1) and IRS2 double knockout (DKO)), metformin action on glycemic control was impaired, which is largely improved by further deletion of hepatic TGF-β1 (TKObeta1) or hepatic Foxo1 (TKOfoxo1). Moreover, blockade of TGF-β1 signaling by chemical inhibitor of TGF-β1 type I receptor LY2157299 improves to metformin sensitivity in mice. Taken together, our current study suggests that hepatic TGF-β1 signaling impairs metformin action on glycemic control, and suppression of TGF-β1 signaling could serve as part of combination therapy with metformin for T2D treatment.

## 1. Introduction

Type 2 diabetes (T2D) is a global epidemic that was affecting more than 460 million individuals as of 2017 [1]. T2D and its complications have caused a tremendous burden due to mortality and disability [2]. The rising burden of T2D is a serious public health concern worldwide. Hyperglycemia is a hallmark of T2D, and the foremost objective for T2D treatment is to maintain the blood glucose level within a normal range [3]. Hepatic glucose production (HGP) accounts for almost 90% of endogenous glucose production and is crucial for the maintenance of systemic glucose homeostasis [4]. Unsuppressed HGP is the main contributor to the development of hyperglycemia in T2D [5]. Targeting dysregulated HGP is the major therapeutic strategy to treat T2D.

Metformin, the current first-line clinical antidiabetic drug, possesses outstanding effectiveness for lowering blood glucose levels [6]. The liver is the major target of metformin to improve blood glucose. Metformin plays a pivotal role in regulating HGP through mitochondrial mechanisms [6]. Metformin triggers inhibition of a specific segment of the mitochondrial electron transport chain—notably, complex I—resulting in an elevated AMP/ATP ratio [6]. This, in turn, activates the energy-sensing enzyme AMPK. Activated AMPK effectively downregulates the expression of glucagon-induced gluconeogenic genes, including *G6pc* and *Pepck* [6,7]. Concurrently, AMPK activation leads to an increase in GLUT4 expression and promotes the translocation of GLUT4 to the cell membrane, resulting in a substantial augmentation of glucose uptake [8,9]. However, with the progression of T2D, most patients develop metformin resistance for glycemic control [10]. The underlying mechanisms of metformin resistance are not well established. Thus, it is urgent to uncover the mechanisms that contribute to the ineffectiveness of metformin in the later stages of diabetes and to develop additional drugs as monotherapy or in combination with metformin to treat T2D.

Transforming growth factor beta 1 (TGF-β1), a member of the TGF-β superfamily, plays a vital role in governing a wide range of cellular processes encompassing cell growth, differentiation, and immune response [11,12,13]. Recent studies revealed the systemic impacts of TGF-β1 on the etiology of T2D [14,15]. Moreover, elevated serum and liver TGF-β1 levels were observed in T2D humans and mice [14,16,17,18]. In hepatocytes, TGF-β1 signaling targets the PP2A-AMPK-Foxo1 axis or the PKA-Foxo1 axis to upregulate *G6pc* expression, promoting HGP [16,17]. Previously, He et al. demonstrated PKA signaling antagonizes metformin action on promoting AMPK activation at the threonine 172 phosphorylation site (pAMPK-T172) via stimulating AMPK phosphorylation at Serine 385 (pAMPK-S485) [19]. Moreover, TGF-β1 suppresses pAMPK-T172 in hepatocytes [16]. These findings suggest potential impacts of TGF-β1 signaling on metformin action in hepatocytes. In this study, we used genetic and chemical strategies to investigate the effect of hepatic TGF-β1 signaling on metformin suppression of HGP and aim to develop new therapeutic strategies in combination with metformin for glycemic control in T2D.

## 2. Results

### 2.1. TGFβ1 Signaling Promotes AMPK Phosphorylation at Serine 485

**Figure 1:**
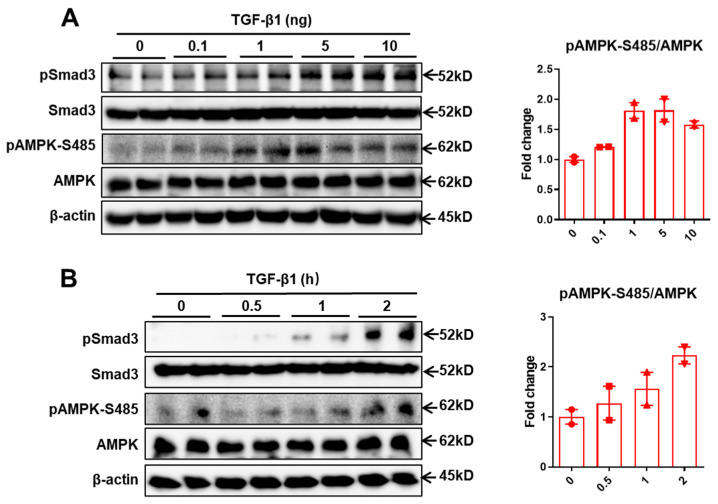
TGF-β1 signaling promotes AMPK phosphorylation at serine 485. (**A**) Western blot of pSmad3, Smad3, pAMPK-S485, AMPK, and β-actin in primary hepatocytes treated with different doses of TGF-β1 for 2 h. (**B**) Western blot of pSmad3, Smad3, pAMPK-S485, AMPK, and β-actin in primary hepatocytes treated with 5ng/mL TGF-β1 for the indicated periods of time.

Previously, He et al. demonstrated that cAMP-PKA signaling antagonizes metformin-stimulated pAMPK-T172 via promoting pAMPK-S485 [19]. Our recent study uncovered TGF-β1-induced activation of PKA signaling in hepatocytes [17]. We first examined the effect of TGF-β1 on pAMPK-S485 in primary hepatocytes. We found that TGF-β1 stimulates pAMPK-S485 levels in primary hepatocytes in a dose- and time-dependent manner (Figure 1A,B). Especially when primary hepatocytes were exposed to higher doses of TGF-β1 (≥5 ng/mL), pAMPK-S485 levels were profoundly increased.

### 2.2. TGFβ1 Signaling Impairs Metformin-Induced AMPK Activation

**Figure 2:**
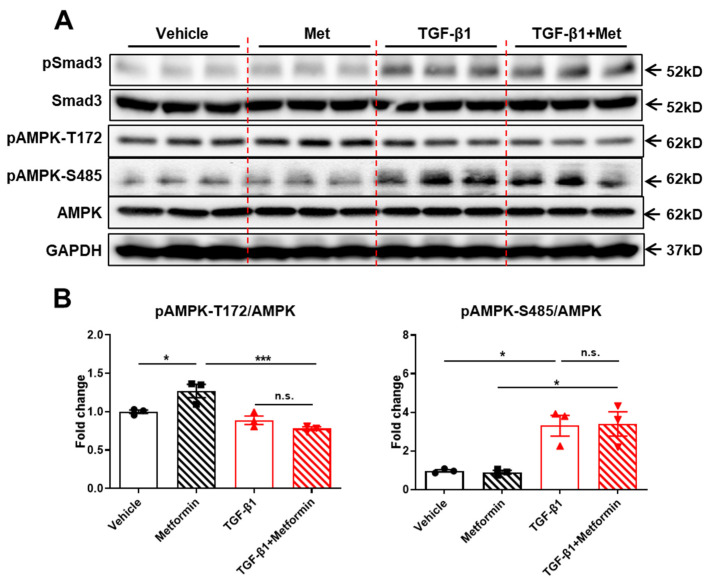
TGF-β1 signaling impairs metformin-induced AMPK activation. (**A**) Western blot of pSmad3, Smad3, pAMPK-S485, AMPK, and GAPDH in primary hepatocytes pretreated with 5 ng/mL TGF-β1 for 1 h and then treated with 100 μM metformin for 30 min. (**B**) Quantification of pAMPK-T172, pAMPK-S485, and AMPK analyzed by ImageJ. Data are presented as mean ± SEM. n.s. (no significance) *p* > 0.05, * *p* < 0.05, *** *p* < 0.001 between assigned groups using one-way ANOVA.

Activation of AMPK at phosphorylation sit T172 (pAMPK-T172) is one of major mechanisms by which metformin suppresses HGP for glycemic control in T2D [6]. However, in T2D, pAMPK-T172 levels are dramatically suppressed [20]. TGF-β1, a cytokine that is positively correlated with body weight and T2D, has been shown to promote HGP [14,16,17,18]. Given that hepatic TGF-β1 signaling promotes AMPK phosphorylation at serine 485 (Figure 1), we were prompted to test whether TGF-β1 affects metformin-induced AMPK activation. As expected, metformin promoted pAMPK-T172 in primary hepatocytes (Figure 2A,B). Such an effect was blocked by TGF-β1 treatment, indicating that TGF-β1 signaling impairs metformin-induced AMPK activation (Figure 2A,B). TGF-β1 significantly stimulated AMPK phosphorylation at serine S485, which was not affected by metformin cotreatment (Figure 2A,B). These results suggest that TGF-β1 signaling impairs metformin-induced AMPK activation at T172 via promoting AMPK-S485 phosphorylation. However, TGF-β1-stimulated pSmad3 levels were unchanged by metformin, suggesting that metformin did not impair TGF-β1 signaling in hepatocytes (Figure 2A).

### 2.3. TGFβ1 Impairs Metformin Action on Suppressing HGP in Primary Hepatocytes

**Figure 3:**
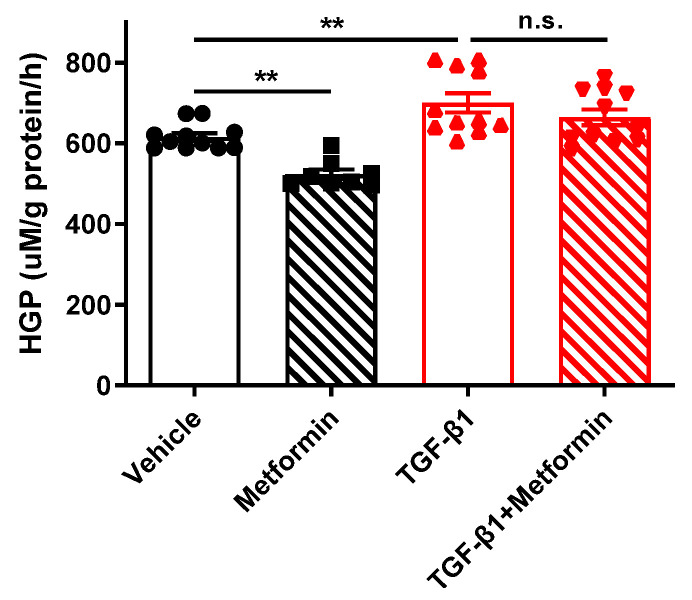
TGF-β1 signaling impairs metformin action on suppressing HGP. Primary hepatocytes were isolated from WT mice and cultured in HGP buffer. Cells were pretreated with 5 ng/mL TGF-β1 for 1 h and then treated with 100 μM metformin for 3 h. HGP buffer was collected to determine glucose production rate. Data are presented as mean ± SEM. n.s. (no significance) *p* > 0.05, ** *p* < 0.01 between assigned groups using one-way ANOVA.

Given the key role of AMPK activation in metformin suppression of HGP, we next investigated whether TGF-β1 signaling affects metformin action on suppressing HGP. Primary hepatocytes isolated from WT mice were cultured in HGP buffer and subjected to indicated treatments. As expected, metformin significantly suppressed HGP, while TGF-β1 treatment significantly promoted HGP (Figure 3). Interestingly, we found that metformin was not able to lower HGP when cotreated with TGF-β1. These results suggest that TGF-β1 signaling impairs metformin action on suppressing HGP in primary hepatocytes.

### 2.4. TGF-β1 Impairs Metformin Suppression of Gluconeogenic Gene Expression in Primary Hepatocytes

**Figure 4:**
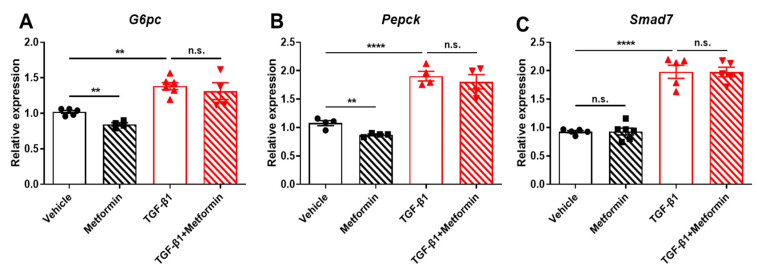
TGF-β1 impairs metformin suppression of gluconeogenic gene expression in primary hepatocytes. Primary hepatocytes were isolated from WT mice and cultured in serum-free DMEM medium. Cells were pretreated with 5 ng/mL TGF-β1 for 1 h and then treated with 100 μM metformin for 3 h. (**A**–**C**) The mRNA was extracted from these cells and then subjected to cDNA synthesis. The mRNA expressions of *G6pc* (**A**)*, Pepck* (**B**), *Smad7* (**C**), and *cyclophilin* were determined by real-time qPCR. Data are presented as mean ± SEM. n.s. (no significance) *p* > 0.05, ** *p* < 0.01, **** *p* < 0.0001 between assigned groups using one-way ANOVA.

Suppressing gluconeogenesis via inhibiting the expression of gluconeogenic genes, including *G6pc* and *Pepck*, is the major mechanism by which metformin suppresses HGP. To further validate the effect of TGF-β1 signaling on metformin suppression of HGP, especially gluconeogenesis, we examined the expression of *G6pc* and *Pepck* in TGF-β1 and metformin-treated primary hepatocytes. Consistent with previous reports, metformin significantly suppressed *G6pc* and *Pepck* expression, while TGF-β1 increased these genes’ expression (Figure 4A,B). Interestingly, TGF-β1 treatment blocked metformin action on suppressing *G6pc* and *Pepck* expression (Figure 4A,B). These results indicate that TGF-β1 impairs metformin suppression of gluconeogenesis via regulating *G6pc* and *Pepck* expression. However, TGF-β1-stimulated *Smad7* mRNA expression was unchanged by metformin, further confirming that metformin did not impair TGF-β1 signaling in hepatocytes (Figure 4C).

### 2.5. Hepatic TGF-β1 Deficiency Improves Metformin Sensitivity for Suppressing HGP

**Figure 5:**
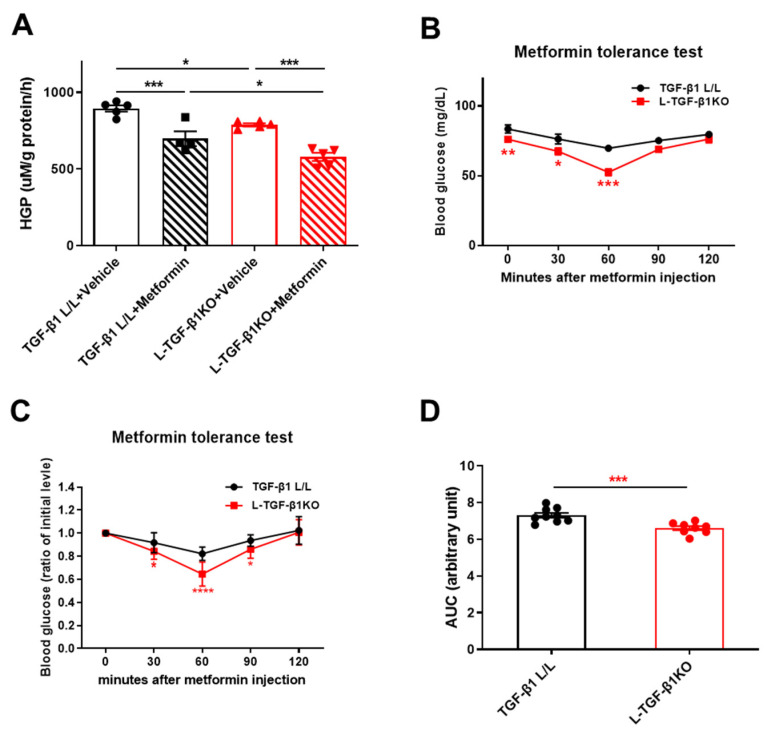
Hepatic TGF-β1 deficiency promotes metformin sensitivity in HFD-induced obese mice. (**A**) Primary hepatocytes were isolated from TGF-β1 L/L and L-TGF-β1KO mice and cultured in HGP buffer. Cells were pretreated with 5 ng/mL TGF-β1 for 1 h and then treated with 100 μM metformin for 3 h. HGP buffer was collected to determine glucose production rate. (**B**,**C**) Metformin tolerance test (actual blood glucose levels (**B**), ratio of initial level (**C**)) in TGF-β1 L/L (*n* = 9) and L-TGF-β1KO (*n* = 8) mice fed with HFD for 3 months. (**D**) Area under the curve of metformin tolerance test shown in (**C**). Data are presented as mean ± SEM. * *p* < 0.05, ** *p* < 0.01, *** *p* < 0.001, **** *p* < 0.0001 between assigned groups or vs. TGF-β1 L/L mice using one-way ANOVA or *t*-test.

We next asked whether TGF-β1 deficiency could improve metformin sensitivity. We generated liver-specific TGF-β1 knockout (L-TGF-β1KO) mice and found that primary hepatocytes isolated from L-TGF-β1KO mice had lower HGP rates compared to those from control (TGF-β1L/L) mice (Figure 5A). Moreover, TGF-β1 deficiency enhanced metformin action on suppressing HGP (22% reduction by metformin in TGF-β1L/L hepatocytes vs. 27% reduction by metformin in L-TGF-β1KO cells) (Figure 5A). Next, we examined whether hepatic TGF-β1 deficiency affects metformin suppression of HGP under pathological condition. We fed L-TGF-β1KO and TGF-β1L/L mice with HFD for 3 months and then performed a metformin tolerance test (MTT) in these mice. We found that L-TGF-β1KO mice displayed lower blood glucose levels after metformin injection compared to those of TGF-β1L/L mice (Figure 5B–D). These results suggest that hepatic TGF-β1 deficiency could improve metformin sensitivity for suppressing HGP.

### 2.6. Hepatic TGF-β1 or Foxo1 Deficiency Restored Metformin Sensitivity in Diabetic Mice

**Figure 6:**
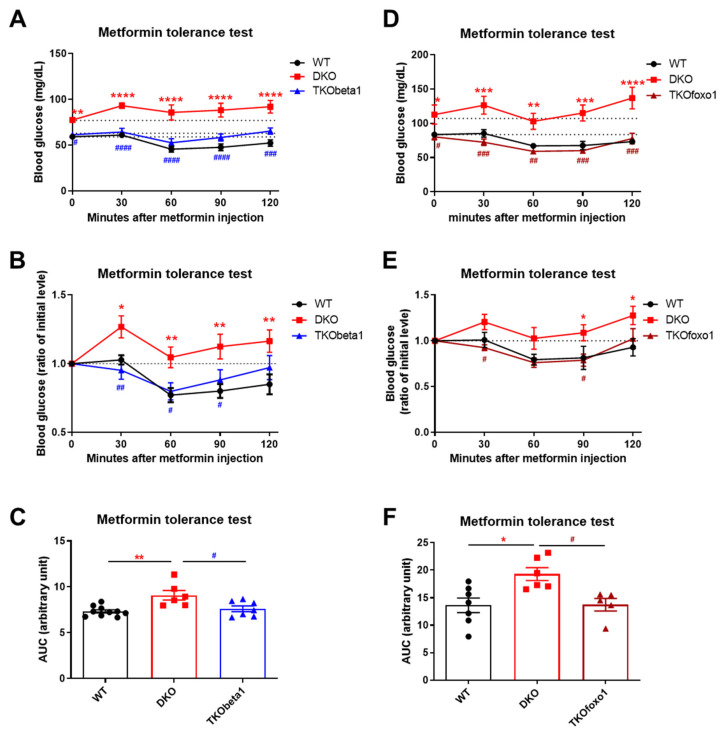
Hepatic TGF-β1 or Foxo1 deficiency restores metformin sensitivity in genetically diabetic mice. (**A**,**B**) Metformin tolerance test (actual blood glucose levels (**A**), ratio of initial level (**B**)) in WT (*n* = 10), DKO (*n* = 6), and TKObeta1 (*n* = 7) mice. (**C**) Area under the curve of metformin tolerance test shown in (**B**). (**D**,**E**) Metformin tolerance test (actual blood glucose levels (**D**), ratio of initial level (**E**)) in WT (*n* = 7), DKO (*n* = 6), and TKOfoxo1 (*n* = 5) mice. (**F**) Area under the curve of metformin tolerance test shown in (**E**). Data are presented as mean ± SEM. * *p* < 0.05, ** *p* < 0.01, *** *p* < 0.001, **** *p* < 0.0001 between WT and DKO, # *p* < 0.05, ## *p* < 0.01, ### *p* < 0.001, #### *p* < 0.0001 between DKO and TKObeta1 or TKOfoxo1 using one-way ANOVA.

Metformin is an insulin sensitizer that enhances liver insulin sensitivity, reducing HGP [21]. Our recent study demonstrated that hepatic TGF-β1 deficiency improves insulin sensitivity in mice [17]. To test whether the beneficial effects of hepatic TGF-β1 deficiency on metformin suppression of HGP are dependent on insulin signaling, we generated liver-specific insulin resistant mice, in which insulin receptor substrate 1 (IRS1) and IRS2 were genetically deleted (DKO mice). These DKO mice displayed diabetic phenotypes that included hyperglycemia, hyperinsulinemia, and severe insulin resistance [17,22,23,24]. Here, we found that DKO mice displayed metformin resistance with barely changed blood glucose levels after metformin administration (Figure 6A,B,D,E), suggesting that metformin lowers blood glucose in a hepatic insulin signaling dependent manner. Surprisingly, further deletion of TGF-β1 in DKO mice (TKObeta1) restored metformin action on suppressing HGP, suggesting that the beneficial effects of TGF-β1 deficiency on metformin suppression of HGP is independent of insulin signaling (Figure 6A–C). Moreover, further deletion of Foxo1 (TKOfoxo1 mice), which has been shown to be a target of TGF-β1 for control of liver metabolism [17,25], also restored metformin sensitivity in DKO mice (Figure 6D–F). Thus, the TGF-β1→Foxo1 axis may serve as a promising target to improve metformin sensitivity for glycemic control.

### 2.7. Pharmacological Inhibition of TGF-β1 Signaling Enhances Metformin Suppression of HGP in Primary Hepatocytes

**Figure 7:**
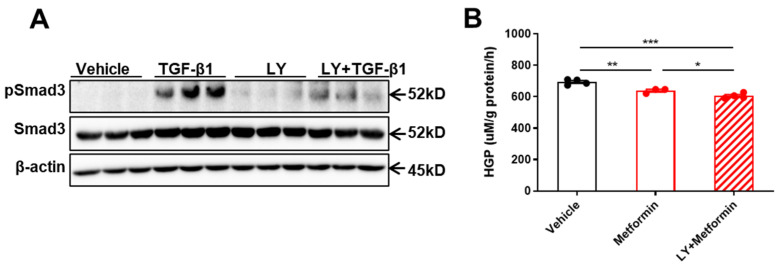
Pharmacological inhibition of TGFβ1 signaling improves metformin sensitivity. (**A**) Primary hepatocytes isolated from WT mice were pretreated with 10 μM LY2157299 (LY) for 30 min and then treated with 5 ng/mL TGF-β1 for 3 h. The pSmad3, Smad3, and β-actin protein levels were determined by Western blot. (**B**) Primary hepatocytes were isolated from WT mice and cultured in HGP buffer. Cells were pretreated with 10 μM LY for 30 min and then treated with 100 μM metformin for 3 h. HGP buffer was collected to determine glucose production rate. Data are presented as mean ± SEM. * *p* < 0.05, ** *p* < 0.01, *** *p* < 0.001, between assigned groups or vs. vehicle group using one-way ANOVA or *t*-test.

In addition to genetic approaches to knockout hepatic TGF-β1, we next tested whether pharmacological inhibition of TGF-β1 signaling could also improve metformin sensitivity. We used LY2157299 (LY), a TGF-β1 receptor I (TβRI) inhibitor, to block TGF-β1 signaling in primary hepatocytes, as indicated by the observation that TGF-β1-stimulated Smad3 phosphorylation was largely impaired by LY (Figure 7A). Interestingly, we found that LY treatment enhanced metformin suppression of HGP (metformin: 638 μM/g protein/h vs. LY+meformin: 605 μM/g protein/h, *p* < 0.05) (Figure 7B).

### 2.8. Pharmacological Inhibition of TGF-β1 Signaling Improves Metformin Sensitivity

**Figure 8:**
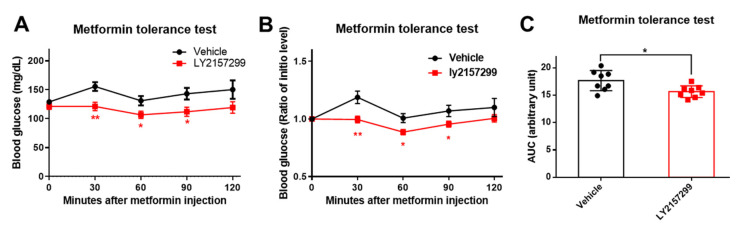
Pharmacological inhibition of TGFβ1 signaling improves metformin sensitivity in HFD-fed mice (*n* = 8/group). (**A**,**B**) Metformin tolerance test (actual blood glucose levels (**A**), ratio of initial level (**B**)) in mice fed with HFD for 3 months with LY2157299 administration for 6 weeks. (**C**) Area under the curve of metformin tolerance test in (**B**). Data are presented as mean ± SEM. * *p* < 0.05, ** *p* < 0.01 between assigned groups or vs. vehicle group using *t*-test.

To further determine the effect of pharmacological inhibition of TGF-β1 signaling on metformin sensitivity under a pathological condition, we fed WT with HFD for 3 months and started to deliver LY2157299 (LY), a TGF-β1 receptor I (TβRI) inhibitor, to these mice for 6 weeks from Week 7. We found that 6 weeks of LY administration significantly enhanced metformin action on suppressing HGP (Figure 8A–C). Consequently, these findings reinforce the pivotal role of TGF-β1 signaling to control metformin action and highlight the potential of TGF-β1 signaling inhibitors in the development of anti-diabetic medications.

## 3. Discussion

**Figure 9:**
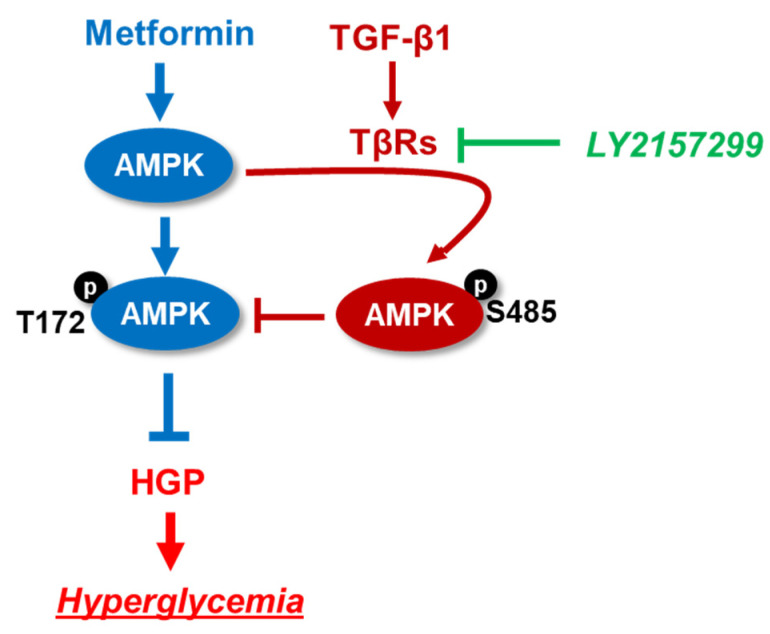
Schematic diagram of the interaction between TGFβ1 signaling and metformin in glycemic control. (1) Metformin suppresses HGP to treat hyperglycemia via promoting AMPK activation at T172 phosphorylation site; (2) TGFβ1 signaling impairs metformin action on AMPK T172 activation via promoting AMPK S485 phosphorylation; (3) Inhibition of TGFβ1 signaling by LY2157299 promotes metformin sensitivity for glycemic control. TβRs: TGF-β1 receptors; →: stimulation; ⊣: inhibition (Blue: The effects of metformin. Green: the effect of LY2157199. Red: the effects of TGF-b1).

In the current study, we firstly provide genetic and chemical evidence that TGF-β1 signaling impairs metformin action in primary hepatocytes and glycemic control in obese and diabetic mice. Our results present three important findings: (1) TGF-β1 treatment impairs metformin-stimulated AMPK activation at T172 via promoting pAMPK-S485 and dampens metformin suppression of HGP; (2) hepatic TGF-β1 deficiency improves metformin sensitivity for lowering blood glucose in HFD-induced obese mice and genetically diabetic mice; (3) pharmacological inhibition of TGF-β1 signaling by the TβRI inhibitor LY2157299 enhances metformin suppression of HGP in primary hepatocytes and obese mice (Figure 9). These results also suggest that hepatic TGF-β1 could serve as potential therapeutic targets to combat the ineffectiveness of metformin in T2D. Drugs inhibiting TGF-β1 signaling can be used in combination with metformin for glycemic control in T2D.

Metformin is widely recognized as the principal therapeutic agent for the management of hyperglycemia among T2D patients [26]. However, a considerable proportion of T2D patients, exceeding one-third of these patients, exhibit either a sluggish or non-existent response to metformin therapy [27]. This phenomenon presents a substantial challenge in diabetes management. Moreover, a notable number of patients experience a progressive decline in their responsiveness to metformin over time, a concern that complicates long-term glycemic control [28]. In the current study, we hypothesize that the elevated TGF-β1 in T2D may dampen the hypoglycemic effects of metformin in the liver. We found that TGF-β1 treatment promotes AMPK phosphorylation at serine 485, which suppresses AMPK activation at T172 in primary hepatocytes (Figure 1). Especially when primary hepatocytes were exposed to high concentration of TGF-β1 (more than 1 ng), pAMPK-S485 was profoundly increased (Figure 1A). Given that TGF-β1 levels were elevated in T2D, the TGF-β1-stimulated pAMPK-S485 potentially contributes to the decreased pAMPK-T172 levels observed in T2D [20]. Moreover, we found that hepatic TGF-β1 deficiency or pharmacological inhibition of TGF-β1 signaling improves metformin suppression of HGP. These findings introduce innovative strategies that aim to circumvent the ineffectiveness of metformin in glycemic control among T2D patients: (1) reduction of the overproduction of TGF-β1 and (2) suppression of the TGF-β1 signaling pathway. Strategies to silence hepatic TGF-β1 expression or drugs antagonizing TGF-β1 signaling could serve as part of combination therapy with metformin to treat T2D.

The liver is the major target of metformin to improve blood glucose levels in T2D [29]. Metformin targets mitochondria to suppress ATP production, which is required for HGP. Metformin inhibits Complex I of the respiratory chain, preventing mitochondrial ATP production, causing increased cytoplasmic ADP:ATP and AMP:ATP ratios, and thus activating AMPK at T172. AMPK-dependent suppression of gluconeogenic gene expression is a major mechanism by which metformin exerts its hypoglycemic effect in the liver [6]. AMPK phosphorylates CREB-regulated transcriptional co-activator-2 (CRTC2), causing CRTC2 to be retained in the cytoplasm, which suppresses the effects of PKA signaling on *G6pc* and *Pepck* expression in the liver [30,31]. AMPK also phosphorylates PDE4B to reduce cellular cAMP levels, thus suppressing gluconeogenesis in hepatocytes [32]. He et al. previously demonstrated that the hyperactivation of cAMP-PKA signaling could antagonize metformin-induced activation of AMPK via promoting AMPK phosphorylation at Ser 485. Here, we found that TGF-β1 signaling promotes pAMPK-S485, blocking metformin induced pAMPK-T172. This potentially contributes to the antagonizing effect of TGF-β1 on metformin suppression of HGP.

Although AMPK plays critical roles in metformin suppression of glucose production, metformin suppresses HGP in both control and AMPK-null hepatocytes [33]. A potential explanation for this is that metformin has an additional AMPK-independent effect by lowering cAMP and reducing the expression of gluconeogenic enzymes [32,34]. Metformin inhibits cAMP-PKA signaling and reduces HGP in primary hepatocytes [7]. In our previous study, we detailed how metformin diminishes glucagon-stimulated HGP through inhibiting the PKA-Foxo1 signaling pathway [7,35]. This discovery was significant for understanding the cellular mechanisms of metformin action and is critical for developing targeted therapies in diabetes management. Moreover, our findings suggest a limited effect of other clinical drugs, like aspirin/salicylate, which also act on similar pathways to suppress HGP, in combination with metformin [7]. This is likely due to their overlapping mechanisms of action, which may not provide additive benefits in glycemic control in T2D. Our recent study demonstrated that TGF-β1 activates PKA-Foxo1 signaling in hepatocytes in a cAMP-independent manner. TGF-β1, without affecting cellular cAMP levels, promotes the binding between PKA and Foxo1 and stimulates Foxo1 activation at Ser 273, promoting HGP [35]. In addition to the pAMPK-S485-dependent suppression of metformin action, TGF-β1 may also dampen metformin effect on PKA signaling to suppress HGP.

TGF-β1 levels are elevated in obesity and T2D humans and mice [14,18]. Yadav et al. has shown protective effects of blockading TGF-β1 by anti-TGF-β1 antibody in obese and diabetic mice [14]. Our recent study suggests that hepatic TGF-β1 deficiency improves glucose and energy metabolism in obesity [17]. Here, we provide evidence that genetic deletion of hepatic TGF-β1 also improves metformin action on glycemic control in obese mice. A number of approved nucleic acid therapeutics are increasing, demonstrating the potential of targeting hepatic TGF-β1 to treat T2D. Currently, small interfering RNA (siRNA) conjugates, lipid nanoparticles (LNPs), and adeno-associated virus vectors (AAVs) are the major technologies of nucleic acid therapeutics [36]. N-acetylgalactosamine (GalNAc)-siRNA conjugates target the asialoglycoprotein receptor (ASGPR), which is predominantly expressed in hepatocytes, and enable hepatic siRNA delivery [36]. LNPs containing siRNA administered intravenously can be internalized by hepatocytes via the low-density lipoprotein receptor (LDLR), which enables its potential for hepatocyte gene silencing. GalNAc-conjugated TGF-β1 siRNA or LNPs containing TGF-β1 siRNA could be potential strategies to reduce the overexpressed hepatic TGF-β1 in obesity and diabetes and to treat T2D in combination with metformin. AAV8 has been shown to effectively deliver genes to the liver of rodents and non-human primates [37,38]. AAV8 expressing short-hair RNA (shRNA) targeting TGF-β1 could also be used to silence TGF-β1 in the liver to combat the ineffectiveness of metformin in T2D over time.

In addition to the induction of TGF-β1 expression by overnutrition or lack of exercise [14,39], genetic polymorphisms of the human TGF-β1 gene have also been shown to correlate with the incidence of obesity and T2D. The TGF-β1 T869C (rs1800470; T29→C) polymorphism, which results in a Leu to Pro substitution at codon 10, is associated with increased abdominal obesity, insulin resistance, T2D, and diabetic nephropathy [40,41]. Another TGF-β1 polymorphism, -C509T (rs1800469), has been shown to be associated with the pathogenesis of T2D [42]. These polymorphisms have been shown to regulate TGF-β1 expression and plasma TGF-β1 levels in humans [43,44]. Further studies investigating whether these genetic alterations in the TGF-β1 gene contribute to the sluggish or non-existent response to metformin therapy may provide new insights for the development of novel strategies to treat diabetes.

The interactions between metformin and TGF-β1 signaling extend beyond glucose regulation. Recent findings indicate that metformin directly interferes with the binding between TGFβ1 and TβRII, thereby further impeding downstream TGFβ signaling pathways [45,46,47]. Studies have indicated that metformin inhibits TGF-β1 signaling pathways, offering potential therapeutic benefits for conditions like fibrosis and cancer [45,48]. This expands the scope of metformin’s utility, making it a versatile tool for managing various pathologies [49]. However, the interactions between metformin and TGF-β1 signaling are diverse and vary depending on the specific tissue and disease context [50]. The intricate mechanisms through which metformin influences TGF-β1 signaling, and the consequent cellular responses, require further investigation.

## 4. Materials and Methods

### 4.1. Animal Experiments

The mouse models utilized in this research included C57BL/6 mice with conditional removal of IRS1/IRS1 genes (DKO) and albumin-Cre mice, both of which have been previously detailed [17]. Floxed TGF-β1 mice (TGF-β1L/L, Jackson Lab, Bar Harbor, ME, USA) were bred with Alb mice (Jackson Lab) to generate liver-specific TGF-β1 knockout mice (L-TGF-β1KO). TGF-β1L/L were bred with DKO mice to create liver-specific IRS1, IRS2, and TGF-β1 triple knockout mice (TKObeta1). Foxo1L/L mice were bred with DKO mice to create liver-specific IRS1, IRS2, and Foxo1 triple knockout mice (TKOfoxo1). This study exclusively used male mice, which were housed in a controlled environment with a temperature of 22 °C and a 12 h light/dark cycle and with unrestricted access to food and water. All procedures involving these animals were conducted in compliance with the ethical guidelines set by the Texas A&M University Institutional Animal Care and Use Committee. The animal study protocol was approved by the Texas A&M University Institutional Animal Care and Use Committee (IACUC 2022-0100, approved on 15 May 2022).

### 4.2. Primary Hepatocytes Isolation and Culturing

Primary mouse hepatocytes were isolated and cultured as previously described [35]. Briefly, mice were infused with a calcium-free HEPES-phosphate buffer I (calcium-free HBSS containing 0.5 mM EGTA and 5.5 mM glucose, 1% penicillin–streptomycin (P/S), pH 7.4). After the color of the liver changed to light brown, collagenase containing buffer II (HBSS with 1.5 mM calcium, 0.5 mg/mL type II collagenase, 5.5 mM glucose, 1% P/S, pH 7.4) was perfused into the liver for digestion. After the appearance of cracking on the surface of the liver, perfusion was stopped, and the liver was excised into ice-cold serum-free DMEM medium. Cells from the digested liver were teased out and suspended in serum-free DMEM medium, filtered through a 70 μm cell strainer, and centrifuged at 1700 rpm for 2 min at 4 °C. The pallet was washed with serum-free DMEM medium twice and mixed with Percoll (adjusted to physiological ionic strength with 10× PBS) to a final concentration of 36% and centrifuged at 1800 rpm for 6 min at 4 °C. After removing the supernatant, the hepatocyte pellet was washed once with serum-free DMEM medium and resuspended in DMEM medium supplemented with 10% fetal bovine serum (FBS) and 1% penicillin–streptomycin (P/S) for 3 h; after cell attachment, hepatocytes were cultured in serum-free DMEM medium overnight and then subjected to treatment for further analysis.

### 4.3. HGP Assay

Primary hepatocytes were freshly isolated from mice and cultured in DMEM supplemented with 2% FBS for 3 h. Subsequently, cells were washed with PBS and cultured in a specifically formulated HGP buffer, which was composed of 118 mmol/L NaCl, 2.5 mmol/L CaCl_2_, 4.8 mmol/L KCl, 25 mmol/L NaHCO_3_, 1.1 mmol/L KH_2_PO_4_, 1.2 mmol/L MgSO_4_, 10 μmol/L ZnSO_4_, 0.6% BSA, 10 mmol/L HEPES, 10 mmol/L sodium DL-lactate, and 5 mmol/L pyruvate and maintained at a pH of 7.4. Cells were subjected to the indicated treatments for the indicated periods of time. HGP buffer cell culture medium was then collected to determine the glucose production rate using the Amplex Red Glucose Assay kit (Invitrogen, Carlsbad, CA, USA). HGP rates were normalized by protein concentration.

### 4.4. Western Blotting

Proteins were prepared from cells or from liver or adipose tissue, resolved by SDS-PAGE, and transferred to nitrocellulose membrane for immunoblotting analysis using specific antibodies as previously described [51]. Briefly, tissue or cells were homogenized in the lysis buffer (25 mM Tris-Cl [pH 7.4], 50 mM sodium pyrophosphate, 100 mM sodium fluoride, 10 mM EDTA, 1% NP-40, 1 mM phenyl-methyl-sulfonyl fluoride, 10 mM sodium orthovanadate, 10 µg/mL aprotinin, and 10 µg/mL leupeptin). Samples were solubilized for 30 min on ice and centrifuged at 12,000× *g* for 15 min at 4 °C. Supernatants were collected, and protein concentrations were determined using the BCA-protein assay (Thermo Fisher Scientific Inc., Rockford, IL, USA). Western blot analyses were performed using 100 µg of tissue lysate or 50 μg of cell lysate. Proteins were resolved by 10% denaturing SDS–polyacrylamide gel and transferred to PVDF membranes. Blots were blocked in BSA-PBST (5% bovine serum albumin (BSA) in phosphate-buffered saline with 0.1% Tween 20) for 1 h and then incubated with primary antibodies in BSA-PBST overnight. Blots were washed with PBST three times (10 min each time) and conjugated to an HRP-coupled secondary antibody before final detection of the conjugate by chemiluminescence (Amersham Biosciences, Little Chalfont, UK). The antibodies used for detecting pAMPK-t172, pAMPK-S485, AMPK-alpha, pSmad3-S423/425, Smad3, GAPDH, and β-actin (Table 1) were acquired from Cell Signaling Technology (Danvers, MA, USA). The signal intensity was measured and analyzed by NIH ImageJ software (version 1.53).

### 4.5. Quantitative Real-Time PCR

The total RNA was extracted from tissue or cells with TRIzol reagent (Invitrogen) and reversely transcribed to cDNA with the iScript cDNA synthesis system (Bio-Rad, Hercules, CA, USA) according to the manufacturer’s instructions. Quantitative gene expression was measured using gene-specific primers (Table 2) using the SYBER Green Supermix system (Bio-Rad) as previously described [51]. The expression of cyclophilin served as the internal control. Gene expression was analyzed with standard ∆∆Ct method of real-time PCR, and the results were presented as the relative fold change of the gene expression compared to that of the control.

### 4.6. Metformin Tolerance Test (MTT)

For the MTT, mice were fasted for 16 h and then received an intraperitoneal injection of either 200 mg/kg metformin (ENZO Life Sciences, Farmingdale, New York, NY, USA) or saline as previously described [7]. Blood glucose levels were measured at specified intervals following metformin injection.

### 4.7. HFD Treatment and LY2157299 Administration

For HFD treatment, mice at the age of 8–10 weeks were fed an HFD (Research Diet, D12492) for 3 months. For LY2157299 administration, LY2157299 (AChemBlock, Hayward, CA, USA) was dissolved in DMSO and then administered (oral gavage) to WT mice at a dosage of 75 mg/kg body weight. This treatment was given daily to mice 6 weeks prior to MTT.

### 4.8. Statistical Analysis

Data in this study were analyzed by GraphPad Prism 9.0 (Boston, MA, USA) and shown as the mean ± standard error of the mean (SEM). To evaluate the statistical significance between two distinct groups, Student’s two-tailed *t*-tests were employed. For comparisons involving multiple groups, either one-way or two-way ANOVA with Tukey post hoc test as appropriate were used. A *p*-value less than 0.05 was deemed to indicate statistical significance.

## 5. Conclusions

TGF-β1 signaling impairs metformin action on glycemic control. Hepatic TGF-β1 deficiency or pharmacological inhibition of TGF-β1 signaling improves metformin sensitivity for control of HGP. However, our current study has a few limitations: (1) Only male mice were used for the in vivo experiment—whether there is sex dimorphism in regulating metformin sensitivity by TGF-β1 signaling is unclear. It will be necessary to consider the use of females in designing experiments to further confirm that the suppression of TGF-β1 signaling could serve as part of combination therapy with metformin for T2D treatment. (2) In our current study, we mainly focus on the effects of LY2157299 and metformin in hepatocytes. Huang et al. demonstrated that metformin preserves beta cell compensation for insulin secretion in prediabetic rats [52]. Amin et al. identified that LY2157299 attenuated GLIS3-deficiency-induced beta cell apoptosis and could serve as a drug candidate for GLIS3-associated diabetes [53]. These results suggest potential interactions between TGF-β1 signaling and metformin in beta cells for the control of blood glucose. Future studies with hepatic TGF-β1 receptor knockout mice will be necessary to validate the hepatic effects of TGF-β1 signaling and metformin in glycemic control. Nevertheless, our current study firstly demonstrated the impact of TGF-β1 signaling on metformin’s regulation of blood glucose and might provide a preclinical test for improving metformin resistance for glycemic control in patients with type 2 diabetes.

## Figures and Tables

**Table 1:** The information about antibodies used in Western blot.

Antibodies	Source	Identifier
pAMK-T172	Cell Signaling Technology	Cat# 2531
pAMK-S485	Cell Signaling Technology	Cat# 2537
AMPK-alpha	Cell Signaling Technology	Cat# 2532
pSmad3-S423/425	Cell Signaling Technology	Cat# 9520
Smad3	Cell Signaling Technology	Cat# 9523
GAPDH	Cell Signaling Technology	Cat# 2118s
β-actin	Cell Signaling Technology	Cat# 4967

**Table 2:** The information about primers used in qPCR.

Genes	Source	Reverse
G6pc forward	Integrated DNA Technologies	CATTGTGGCTTCCTTGGTCC
G6pc reverse	Integrated DNA Technologies	GGCAGTATGGGATAAGACTG
Pck1 forward	Integrated DNA Technologies	CCATCGGCTACATCCCTAAG
Pck1 reverse	Integrated DNA Technologies	GACCTGGTCCTCCAGATA
Smad7 forward	Integrated DNA Technologies	GGCCGGATCTCAGGCATT
Smad7 reverse	Integrated DNA Technologies	TTGGGTATCTGGAGTAAGGAGG
Cyclophilin forward	Integrated DNA Technologies	ACTGAATGGCTGGATGGCAAG
Cyclophilin reverse	Integrated DNA Technologies	TGCCCGCAAGTCAAAAGAAAT

## Data Availability

The data presented in this study are available on request from the corresponding author.
